# Functional Characterization of an Immortalized Human Muscle Cell Line Carrying the Heterozygous RyR1 p.T4288_A4290dup Associated With Rhabdomyolysis

**DOI:** 10.1155/humu/8852225

**Published:** 2026-10-27

**Authors:** Wen Jin, Irem Huriye Ceren, Kenshiro Kido, Alexis Ruiz, Teresa Ianiri, Anna Sarkozy, Francesco Muntoni, Kamel Mamchaoui, Katarzyna Buczak, Francesco Zorzato, Susan Treves

**Affiliations:** ^1^ Departments of Neurology and Biomedicine Basel University Hospital Basel Switzerland unispital-basel.ch; ^2^ Department of Anesthesiology and Critical Care Hiroshima University Hospital Hiroshima Japan hiroshima-u.ac.jp; ^3^ Dubowitz Neuromuscular Center Developmental Neurosciences Research and Teaching Department Great Ormond Street Institute of Child Health University College London London UK ucl.ac.uk; ^4^ NIHR Great Ormond Street Hospital Biomedical Research Center London UK; ^5^ Genetic Therapy Accelerator Center Institute of Neurology and UCL Great Ormond Street Institute of Child Health London UK; ^6^ Institut de Myologie Centre de Recherche en Myologie Université Sorbonne Inserm Paris France inserm.fr; ^7^ Proteomics Core Facility Biozentrum University of Basel Basel Switzerland unibas.ch; ^8^ Department of Life Sciences and Biotechnology University of Ferrara Ferrara Italy unife.it

## Abstract

The ryanodine receptor 1 plays an essential role in skeletal muscle by releasing the calcium necessary for contraction from the sarcoplasmic reticulum and mutations in *RYR1*, the gene encoding the ryanodine receptor 1 are associated with a variety of neuromuscular phenotypes, ranging from core myopathies and centronuclear myopathy to the pharmacogenetic disorder malignant hyperthermia. The muscle phenotype of the patient mainly depends on whether the mutations are dominant or recessive and on their location within the gene. Gain of function *RYR1* mutations usually affect the biophysical properties of the calcium channel and are associated with malignant hyperthermia susceptibility trait with or without a myopathy. From a functional point of view, these mutations render the calcium channel oversensitive thereby facilitating its opening and allowing more calcium to be released from the sarcoplasmic reticulum. More recently exertional rhabdomyolysis has been observed in particularly athletic individuals who also carry gain of function *RYR1* variants, but so far no functional studies have been performed investigating if and how such variants affect ryanodine receptor 1 calcium channels. In the present report we used an immortalized muscle cell line from an individual with exertional rhabdomyolysis carrying the heterozygous RyR1 duplication p.T4288_A4290dup in exon 91 and show that the presence of this variant is associated with morphometric changes in myotubes, enhanced myotube maturation and enhanced calcium release following membrane depolarization and pharmacological RyR1 activation. The enhanced calcium release would lead to more efficient muscle contraction but would also result in increased activation of SERCA pumps, more ATP consumption and increased enzymatic activity of the calcium dependent protease calpain 3. We hypothesize that these events are potentially the underlying (co)causes of exercise‐induced rhabdomyolysis in individuals carrying this RyR1 variant.

## 1. Introduction

Rhabdomyolysis or skeletal muscle fiber breakdown is a potentially life‐threatening event, characterized by the acute elevation of serum creatine kinase, myoglobin, lactate dehydrogenase, and electrolytes, which if left untreated, can lead to acute kidney injury, compartment syndrome, disseminated intravascular coagulation, cardiac dysrhythmias, and death in 10% of the cases. Triggers include exposure to toxins and drugs such as statins, trauma, viral infections, physical activity, metabolic abnormalities, and inherited muscle diseases. An elevation of serum creatine kinase (CK) to levels > 5000 IU/L is the most sensitive laboratory test for evaluating rhabdomyolysis [1, 2].

Because of its diverse triggers, the pathophysiology of rhabdomyolysis is not always clear; nevertheless, the final outcome is characterized by the loss of skeletal muscle integrity and the failure of the muscle cell′s energy supply. At rest ionic pumps including the Na^+^/K^+^ ATPase and Ca^2+^ATPases maintain the appropriate ionic balance within muscle cells, keeping the intracellular Na^+^ and Ca^2+^ concentrations low and the intracellular K^+^ concentration high. An increase in the intracellular Na^+^ concentration brought about by membrane damage or ATP depletion would be accompanied by the influx of water from the extracellular environment further disrupting the intracellular ionic composition and cellular integrity. A sustained increase in cytosolic Ca^2+^ would lead to myofibrillar contraction, activation of Ca^2+^‐dependent proteases and continuous activation of Ca^2+^ pumps leading to ATP depletion. If such conditions persist in time, the final result is myofiber death and a strong inflammatory reaction characterized by the presence of pro‐inflammatory cytokines and release of muscle contents into the bloodstream.

Indeed, patients with muscular dystrophies may develop rhabdomyolysis because of the lack of sarcolemmal structural integrity that leads to a massive influx of Ca^2+^ ions into the muscle fibers [3]. Among inherited metabolic disorders, McArdle disease, carnitine palmitoyltransferase II deficiency and other myopathies related to glycogen metabolism have been linked to a predisposition to develop exercise‐induced rhabdomyolysis most likely because of an impairment in ATP production [4–6]. Interestingly, patients with disorders in skeletal muscle calcium regulation or defects in excitation‐contraction coupling (ECC) also have a predisposition to develop rhabdomyolysis triggered by exercise, heat or environmental causes such as stress or viral infections ([7–11]). ECC is the key process underlying skeletal muscle contraction, whereby an electrical signal (depolarization of the plasmalemma) sensed by the dihydropyridine receptor (DHPR) voltage gated Ca^2+^ channel is converted into a chemical signal (Ca^2+^ release from the sarcoplasmic reticulum, SR) via the opening of the skeletal muscle ryanodine receptor Ca^2+^ channel (RyR1) [12, 13]. Muscle relaxation is brought about by the active pumping of Ca^2+^ back into the SR by the SERCA calcium pumps (SERCA1 and SERCA2) [14, 15]. Patients with pathogenic variants in genes encoding ECC proteins, including gain of function pathogenic variants in *RYR1*, in *ATP2A1* encoding SERCA1 (Brody′s disease) and in *ATP2A2*, encoding SERCA2, have been reported to develop adult‐onset rhabdomyolysis following exercise [7, 10, 16, 17]. In the past decade studies have revealed that *RYR1* pathogenic variants are present in about 30% of the subjects with exertional rhabdomyolysis (ERM), many of whom are athletes or semiprofessional athletes who are unaware of their genetic status [10]. ERM has also been linked to malignant hyperthermia susceptibility (MHS) [8], a pharmacogenetic disorder caused by gain of function *RYR1* pathogenic variants in more than 50% of the patients [18, 19]. In MHS, the pathogenic variants in RyR1 render the Ca^2+^‐release channel hypersensitive to activation, leading to a hypermetabolic reaction after contact with trigger agents such as halogenated anesthetics and the muscle relaxant succinylcholine [18, 19]. Thus, both MHS and ERM are linked to a dysregulated rise in the intracellular Ca^2+^ concentration.

Many aspects of ERM remain obscure, in particular whether there are ties between MHS and ERM, and whether there are features characteristic to muscles of patients with ERM carrying pathogenic *RYR1* variants. In the present study we aimed to answer these questions by investigating the cellular, biochemical, and physiological properties of an immortalized human muscle cell line generated from an individual with ERM harboring the heterozygous RyR1 p.T4288_A4290dup (*RYR1* c.12861_12869dup CACGGCGGC) in exon 91, a variant that has been previously described in several patients with ERM mostly in combination with additional RyR1 variants [7, 20, 21]. Our results show that myotubes from the ERM subject exhibit an altered cellular morphology, with a decreased number of and smaller size of myonuclei, an increased expression levels of the late myogenic maturation marker *TNNT3*. ERM myotubes also exhibited a higher expression level of the *RYR1* transcript and, importantly showed increased Ca^2+^ release following RyR1 activation. Calpain activity was increased following membrane depolarization in myotubes from the individual carrying the RyR1 p.T4288_A4290dup, but not in control myotubes. However, mass spectrometry analysis of mature muscle fibers from three subjects with ERM carrying the same *RYR1* duplication, revealed few overall differences in protein composition compared to muscle biopsies from controls, even though the ERM group and control group were clearly distinct. The results of this study point to a particular effect of the heterozygous RyR1 p.T4288_A4290dup variant on calcium homeostasis in muscles from ERM subjects, with effects on the maturation status of muscle cells, on ECC and on the activity of the calcium‐dependent protease calpain. Such events are more evident in the immature myotube cultures than in mature muscle fibers.

## 2. Materials and Methods

### 2.1. Compliance With Ethical Standards

All procedures were performed in accordance with the ethical standards of the institutional and/or national research committee and with the 1964 Helsinki declaration and its later amendments or comparable ethical standards. This study was approved by the Ethikkommission Nordwest‐ und Zentralschweiz (Permit No. EKNZ 2014‐065); all subjects gave written informed consent to carry out this work.

### 2.2. Muscle Biopsies and Cell Cultures

Quadriceps muscle biopsies from three male subjects aged 12, 13, and 17 years with clinically confirmed ERM (CK levels > 10000 iU/L) were genetically investigated, their *RYR1* was sequenced and all three confirmed to carry the heterozygous RyR1 p.T4288_A4290dup (*RYR1* c.12861_12869dup CACGGCGGC) variant in exon 91. The individual from whom the immortalized muscle cell line was generated (index case or Pt ERM_01) did not carry any other RyR1 variants. The donor of the second biopsy (Pt ERM_02) additionally carried the heterozygous p.H3981Y (*RYR1* c.11941 C>T) benign variant in exon 87, and the donor of the third biopsy (Pt ERM_03) harbored the RyR1 p.T4288_A4290dup plus p.S1342G (c.4024A>G) in exon 28 plus p.R2084Q (c.6251G>A) in exon 40. Muscle biopsies from male age‐matched controls were used for mass spectrometry.

Immortalized myoblast cell lines were established from myoblasts explanted from the quadriceps muscle biopsy of two healthy control donors (cell line N° 3373, or CTRL 1 and cell line 567A or CTRL 2) with no neuromuscular disorders and from the index patient Pt ERM_01 (Family 10 II.1 [7]) carrying the heterozygous RyR1 p.T4288_A4290dup variant (cell line 8191 or ERM), by double transfection with recombinant retroviruses containing the telomerase (hTERT) cDNA and Cdk4 cDNA, as previously described [22, 23]. Immortalized myoblasts were maintained in skeletal muscle cell growth medium (PromoCell GmbH, Heidelberg, Germany; catalogue N° c‐23060) in low oxygen atmosphere (5% O_2_ and 5% CO_2_) at 37° C. In order to induce differentiation, once the density had reached approximately 80%, cells were rinsed one time with phosphate buffered saline (PBS, pH 7.2; Life Technologies) and incubated with skeletal muscle differentiation medium (high glucose DMEM (4.5 mg/mL, ThermoFisher), containing 10 ng/mL insulin and 50 *μ*g/mL gentamicin). The process of differentiation took an average of 5–7 days after which multinucleated myotubes were clearly visible under low magnification.

### 2.3. Immunofluorescent Microscopy, Myotube Morphology, and Fusion Index

Muscle cells were cultured on laminin‐coated Ibidi 0.17 mm *μ*‐Slide 4 well glass bottom slides (Ibid Cat # 80427) until 90% confluent. The medium was switched to differentiation medium for 7 days after which cells were fixed in 4% paraformaldehyde in PBS and visualized using a Nikon Ti2 widefield microscope equipped with a Nikon CFI Plan Apo Lambda 20x air objective (*N* *A* = 0.75). Multichannel fluorescent images were acquired with a Photometrics Prime 95B sCMOS camera, and image acquisition was controlled via Nikon NIS‐Elements software.

For myotube size and nuclear number, fixed cells were stained with 4 ^′^,6‐diamidino‐2‐phenylindole (DAPI) to visualize nuclei and with Phalloidin‐iFluor 647 (Abcam Catalog N° ab 176759) to label the actin cytoskeleton. For each sample, high‐resolution images were obtained using a 4 × 4 tile scan covering the entire culture area, followed by automatic stitching to generate composite images and minimize sampling bias. For each cell line, 6–8 wells containing independently cultured cells were analyzed. Within each well, 15–25 myotubes were randomly selected for morphological analysis. Myotubes with substantial overlap or excessive thickness that prevented reliable delineation were excluded from the analysis. Image analysis was performed using QuPath software (Version 0.5.0.) [24]. Approximately 10 multinucleated myotubes per image were selected from nonoverlapping regions for analysis, excluding areas where myotubes overlapped or formed excessively thick structures. Myotubes were manually delineated to ensure accurate segmentation, and morphological parameters, including myotube area and width, were quantified automatically using QuPath. Nuclear segmentation was performed to measure nuclear size and to determine the number of nuclei per myotube. The fusion index was defined as the percentage of nuclei located within multinucleated myotubes (≥ 3 nuclei) relative to the total number of nuclei.

### 2.4. Intracellular Calcium Measurements

Muscle cells were cultured on laminin‐coated 0.17 mm glass cover slips as previously described [25–27]. Fully differentiated myotubes were loaded with 5 *μ*M fura‐2/AM (Calbiochem; 344905) for 30 min at 37°C and mounted on a temperature‐controlled stage (TempControl 37‐2 digital). The chamber was continuously perfused with Krebs–Ringer medium containing 2 mM Ca^2+^ and cells were stimulated using a 12‐way 100 mm diameter quartz micro‐manifold computer‐controlled microperfuser (ALA Scientific Instruments), as described previously [25, 26]. Recordings were done on a fluorescent Axiovert S100 TV inverted microscope (Carl Zeiss) equipped with a 20x water‐immersion FLUAR objective (NA 0.17) and filters (BP 340/380, FT 425, BP 500/530) attached to a Photometrics Cascade 128+ CCD (charge‐coupled device) camera controlled by VisiView software Version 2.0.8. Changes in the free cytosolic calcium concentration were analyzed using MetaMorph (molecular devices) imaging system and the average pixel value for each cell was measured as previously described.

### 2.5. Calpain Expression and Enzymatic Activity in Myotubes Subjected to Membrane Depolarization and Heat Stress

The human skeletal muscle cell lines were cultured and differentiated into mature myotubes over 7–10 days. On the experimental day, myotubes were preincubated for 5 min at either 37°C (physiological temperature) or 42°C (heat stress). Subsequently, membrane depolarization was induced by stimulating the cells with 60 mM KCl for 5 min after which the solution was rapidly aspirated, replaced with fresh differentiation medium and the cells were returned to the incubators for 0, 60, 120, and 240 min. Experimental controls were incubated in Krebs–Ringer solution for 5 min rather than with KCl. At each time point, cell pellets were collected for RNA isolation and used for qPCR analysis of transcript expression.

Calpain activity was assessed using the Calpain Activity Assay Kit (Abcam Catalog N° ab65308), according to the manufacturers′ recommendations. Following myotube depolarization induced by the addition of 60 mM KCl, cells were washed with ice‐cold PBS and scraped in the extraction buffer provided in the kit. The total protein concentration in the cell extracts was determined using the Bio‐Rad Protein Assay (Bio‐Rad, 500‐0006). For each enzymatic assay 100 *μ*g of total protein was used per sample. Measurements were performed in duplicate, and calpain activity was expressed as relative fluorescence units (RFU).

### 2.6. Quantitative PCR

Was performed as previously described [28]. Briefly, RNA was isolated from myotubes using TRIzol reagent (Thermo Fischer; 15596026) following the manufacturer′s protocol. For subsequent quantitative real‐time polymerase chain reaction (qPCR), 1000 ng of RNA was reverse‐transcribed to cDNA using the High‐capacity cDNA Reverse Transcription Kit (Applied Biosystems; 4368814) on an Applied Biosystems 2720 Thermal Cycler. The cDNA was amplified using PowerUP Sybr Green Master Mix (Applied Biosystems; A25742) for regular DNA quantification on an Applied Biosystems 7500 Fast Real‐time PCR System running 7500 software Version 2.3. A complete list of the transcripts whose expression was investigated and the primers used is shown in Table 1.

**Table 1:** Differentially expressed proteins in muscle biopsies of ERM versus control individuals.

Gene name	Protein name	Fold change	Q value	Log10Q
RBM3	RNA‐binding protein 3	−2.43879	0.00093	3.031609
IGFN1	Immunoglobulin‐like and fibronectin Type III domain‐containing protein 1	−2.19523	0.007193	2.143069
ACTG1	Gamma actin	−2.03089	0.027921	1.554075
SLC44A1	Choline transporter‐like protein 1	−1.98405	0.023845	1.62261
S100A16	S100 A16 protein	−1.97192	0.030468	1.51616
CORO1A	Coronin 1A	−1.6743	0.000497	3.303278
PECAM1	Platelet endothelial cell adhesion molecule 1 (CD31)	−1.6483	0.003361	2.473566
CFHR1	Complement factor H‐related 1	−1.62723	0.004644	2.333079
C4BPA	Complement component 4 Binding protein alpha	−1.62314	0.012508	1.902829
FERMT3	Fermitin family homolog 3 or kindlin‐3, MIG2‐like protein	−1.59628	0.002703	2.568192
PDGFRB	Platelet derived growth factor receptor beta	−1.54226	0.006766	2.169658
EZR	Ezrin	−1.52921	0.007101	2.148678
PLEK	Pleckstrin	−1.52086	0.017836	1.748711
PON1	Paraoxonase 1	−1.49827	0.049534	1.305099
TMEM43	Transmembrane protein 43	−1.47798	0.031238	1.505323
RAB8A	Rab8A	−1.41361	0.043224	1.36427
ACLY	ATP citrate lyase	−1.40786	0.041521	1.381729
CILP	Clip protein	−1.40449	0.000669	3.174811
PPBP	Proplatelet basic protein	−1.39184	0.034002	1.468499
TMEM258	Transmembrane protein 258	−1.31761	0.010216	1.990732
HPRT1	Hypoxanthine phosphoribosyltransferase 1	−1.31272	0.025747	1.589273
GIMAP4	GTPase IMAP family member 4	−1.30494	0.035206	1.453385
KYAT3	Kynurenine aminotransferase 3	−1.28225	0.019274	1.715038
OSTC	Olygosaccharyltransferase complex noncatalytic subunit	−1.28122	0.015861	1.799669
SCAMP3	Secretory carrier membrane Protein 3	−1.26485	0.009987	2.000582
GSN	Gelsolin	−1.23766	0.019905	1.701045
SEPTIN8	Septin 8	−1.12976	0.018214	1.739599
HSPA12B	Heat Shock protein family A (HSP70) member 12B	−1.11847	0.005375	2.26965
NIBAN2	Niban apoptosis regulator 2	−1.11317	0.011161	1.952312
ABCC1	Multidrug resistance‐associated protein 1	−1.09827	0.003101	2.508511
CIRBP	Cold‐inducible RNA‐binding protein	−1.07422	0.043835	1.358183
CYB5R3	Cytochrome b5 reductase 3	−1.03251	0.003015	2.52069
GADD45GIP1	Growth arrest and DNA‐damage‐inducible proteins‐interacting protein 1	−1.00898	0.038486	1.414693

### 2.7. Western Blotting

Total homogenates of myotubes were prepared as previously described [25]. Protein concentration was determined using the Bradford protein assay kit (Bio‐Rad, 500‐0006) using bovine serum albumin as a standard. Proteins (30 *μ*g/lane) were separated on 6% SDS‐PAGE and blotted onto nitrocellulose. For immunoblotting, membranes were blocked for 60 min at room temperature or overnight at 4°C in 1% blocking buffer (Roche; 11500694001) and incubated with rabbit monoclonal anti‐RyR1 (Cell Signaling, N° 8153S) antibody or antimyosin heavy chain all (Millipore, N° 05‐716). Secondary antibodies/proteins used were either antimouse peroxidase (Sigma, N°A2304) or protein‐G peroxidase (Sigma, N° P8170). Bands were visualized using the SuperSignal Dura West kit (Thermo Scientific; 34076). For quantification, films were scanned on an Epson Perfection V700 Photo as 16‐bit tiff image and densitometrically analyzed using the GelAnalyzer software Version 2010a.

### 2.8. Myoblast Proliferation Index

Myoblast proliferation was assessed using the CellTiter 96 AQueous One Solution Cell Proliferation Assay (MTS) (Promega Catalog N° G3582). To ensure optimal signal detection within the linear range, a seeding density of 4 × 10^4^ cells per well was used. Technical duplicates were plated in 96‐well plates and incubated for 0 h (T_0_) and 24 h (T_24_). Following the addition of the MTS reagent, the absorbance was measured at 490 nm using a Synergy H1 Hybrid Multi‐Mode Microplate Reader (BioTek Instruments, Winooski, Vermont, United States). The proliferation index was calculated as the ratio of the optical density (OD) at 24 h to the OD at 0 h (OD T_24_/OD T_0_).

### 2.9. LC‐MS Analysis

Muscle biopsies were processed for mass spectrometry as previously described [29] starting from approximately 10 mg of tissue that had been stored in liquid N_2_. Briefly, muscles were solubilized in a lysis buffer (5% SDS, 10 mM TCEP, 0.1 M TEAB) and lysed by sonication using a PIXUL Multi‐Sample Sonicator (Active Motif) with Pulse set to 50, PRF to 1, process time to 10 min and burst rate to 20 Hz. Lysates were incubated for 10 min at 95°C, alkylated in 20 mM iodoacetamide for 30 min at 25°C and proteins digested using S‐Trap micro spin columns (Protifi) according to the manufacturer′s instructions. Shortly, 12% phosphoric acid was added to each sample (final concentration of phosphoric acid 1.2%) followed by the addition of S‐trap buffer (90% methanol, 100 mM TEAB pH 7.1) at a ratio of 6:1. Samples were mixed by vortexing and loaded onto S‐trap columns by centrifugation at 4000 g for 1 min followed by three washes with S‐trap buffer. Digestion buffer (50 mM TEAB pH 8.0) containing sequencing‐grade modified trypsin (1/25, w/w; Promega, Madison, Wisconsin) was added to the S‐trap column and incubate for 1 h at 47°C. Peptides were eluted by the consecutive addition and collection by centrifugation at 4000 g for 1 min of 40 ul digestion buffer, 40 *μ*L of 0.2% formic acid and finally 35 *μ*L 50% acetonitrile, 0.2% formic acid. Samples were dried under vacuum and stored at −20°C until further use.

Dried peptides were resuspended in 0.1% aqueous formic acid, 0.02% DDM (n‐Dodecyl‐B‐D‐maltoside) and 25 ng of peptides were subjected to LC–MS/MS analysis using a timsTOF Ultra Mass Spectrometer (Bruker) equipped with a CaptiveSpray nano‐electrospray ion source (Bruker) and fitted with a Vanquish Neo (Thermo Fisher Scientific). Peptides were resolved using a RP‐HPLC column (100 *μ*m × 30 cm) packed in‐house with C18 resin (ReproSil Saphir 100 C18, 1.5 *μ*m resin; Dr. Maisch GmbH) at a flow rate of 0.4 *μ*l/min and column heater set to 60°C. The following gradient was used for peptide separation: from 2% B to 25% B over 25 min to 35% B over 5 min to 95% B over 1 min followed by 5 min at 95% B to 2% B over 1 min followed by 3 min at 2% B. Buffer A was 0.1% formic acid in water and buffer B was 80% acetonitrile, 0.1% formic acid in water.

The mass spectrometer was operated in dia‐PASEF mode with a cycle time estimate of 0.96 s. MS1 and MS2 scans were acquired over a mass range from 100 to 1700 m/z. A method with 8 dia‐PASEF scans separated into three ion mobility windows per scan covering a 400–1000 m/z range with 25 Da windows and an ion mobility range from 0.64 to 1.37 Vs cm^2^ was used. Accumulation and ramp time were set to 100 ms, capillary voltage was set to 1600 V, dry gas was set to 3 l/min and dry temperature was set to 200°C. The collision energy was ramped linearly as a function of ion mobility from 59 eV at 1/K0 = 1.6 Vs cm^−2^ to 20 eV at 1/K0 = 0.6 Vs cm^−2^ [30].

The acquired files were searched using the Spectronaut (Biognosys v20.3) with a default setting of a directDIA workflow, against a Homo Sapiens database (consisting of 20360 protein sequences downloaded from Uniprot on 20220222) and 393 commonly observed contaminants. Quantitative fragment group data (FG.MS2RawQuantity) was exported from Spectronaut and processed using ProteoFlux v1.8.8 (10.5281/zenodo.18640999). To meet additional assumptions (normality and homoscedasticity) underlying the use of linear regression models and *t*‐tests, intensity signals were transformed from the linear to the log‐scale. Data was normalized by median_equalization, imputed using the lc_conmed method and *p* − values for pairwise comparisons were calculated using the limma package (doi:10.18129/B9.bioc.limma). The resulting per protein and condition comparison *p* − values were adjusted for multiple testing using the Benjamini–Hochberg method. Principal component analysis was performed using only proteins quantified in all six samples, without missing value imputation. For rank‐based enrichment analysis, proteins were ranked according to their calculated log2FC values and analyzed using the STRING database to identify enriched functional clusters among proteins increased or decreased in the ERM group.

### 2.10. Statistical Analysis

Statistical analyses were performed using GraphPad Prism (Version 11). All values show the mean ± SEM unless otherwise stated. Calcium dose–response curves were fitted by nonlinear regression using the Origin software to determine half‐maximal effective concentration (EC50) values. The values for resting fura‐2 fluorescence, total releasable calcium from intracellular stores, peak KCl and 4‐cmc were compared using one‐way ANOVA followed by an uncorrected Fisher′s LSD for post hoc analysis. For myoblast proliferation Kruskal–Wallis test was followed by an uncorrected Dunn′s multiple comparisons test for post hoc analysis. For myotube morphology and fusion index one‐way ANOVA followed by Tukey′s multiple comparisons test and adjusted *p* values are indicated. For myotube maturation markers and ECC transcript analysis Kruskal–Wallis test followed by Dunn′s multiple comparisons test for post hoc analysis and adjusted *p* values are indicated. Differences were considered statistically significant when *p* < 0.05.

## 3. Results

### 3.1. Myotubes Immortalized From the Individual Carrying the RyR1 c.T4288_A4290dup Variant Are Morphologically Different and More Mature, Compared With Myotubes Immortalized From Healthy Controls

In order to investigate the impact of the RyR1 variant c.T4288_A4290dup on the cellular, biochemical and physiological aspects on muscle function, we first investigated whether the myotubes carrying the RyR1 variant linked to ERM are morphologically different from myotubes derived from two healthy controls, by evaluating myotube size, nuclear size, number of nuclei per myotube and fusion index. A total of 125 myotubes were analysed from 6–8 wells (cells were plated in 6–8 different wells on different days) for CTRL1, 155 myotubes were analyzed from 6‐8 wells for CTRL2 and 183 myotubes were analyzed from 6–8 wells for ERM. High‐resolution images on myotubes stained with DAPI and phalloidin were obtained as described in the Methods. Figure 1A shows DAPI stained (left panels), phalloidin (central panels), and merged (right panels) images of myotubes from CTRL1 (top), CTRL2 (central), and the ERM individual (bottom) whereas Figure 1B shows the morphometric evaluations. Each symbol represents measurements performed on a single myotube or nucleus. Myotubes from Pt ERM_01 carrying the RyR1 variant were 68.8% thinner (the mean myotube diameter was 33.9 *μ*m *n* = 183 for ERM, 47.5 *μ*m *n* = 125 for CTRL1 and 51.1 *μ*m *n* = 155 for CTRL2; adjusted *p* value < 0.0001) and their surface area was 54.0% of that of control myotubes (the mean area was 6480 *μ*m^2^ for ERM, 11254 *μ*m^2^ for CTRL1, and 12723 *μ*m^2^ for CTRL2 myotubes; adjusted *p* value < 0.0001). In addition, the nuclear area of ERM myotubes was 80.4% of that of control myotubes (the mean area was 101.7 *μ*m *n* = 2230 for ERM, 129.1 *μ*m^2^ *n* = 1694 for CTRL1 and 123.9 *μ*m^2^ *n* = 2592 for CTRL2 myotubes; adjusted *p* value < 0.0001) and the average number of nuclei per ERM myotube was 79.0% of that of control myotubes (14.1 *n* = 183 for ERM, 17.0 *n* = 124 for CTRL1 and 18.7 *n* = 156 for CTRL2 myotubes; adjusted *p* value < 0.0001). It should be pointed out that CTRL1 and CTRL2 myotubes also showed morphometric variability but the extent was much smaller (between 0.5% and 6%) than that shown by ERM and control myotubes (Figure 1B). Interestingly, the fusion index, defined as the percentage of nuclei located within multinucleated myotubes (≥ 3 nuclei) relative to the total number of nuclei in myotubes derived from the ERM, CTRL1, and CTRL2 individuals, was similar (mean ± SEM fusion index was 0.55 ± 0.03*n* = 34, 0.52 ± 0.03*n* = 23 and 0.52 ± 0.03*n* = 20 in ERM, CTRL1, and CTRL2 myotubes, respectively; Figure 1C). The proliferation rate of myoblasts from the ERM individual was significantly reduced compared with that from the two control cell lines (the mean ± SEM proliferation index was 1.48 ± 0.16*n* = 7, 2.13 ± 0.17*n* = 7, and 2.20 ± 0.25*n* = 7 for ERM, CTRL1, and CTRL2 myoblasts, respectively, *p* = 0.0058 and *p* = 0.0051 for ERM versus CTRL1 and CTRL2; Kruskal–Wallis test followed by an uncorrected Dunn′s multiple comparison test for post hoc analysis) whereas the myoblast proliferation of the two control cell lines was similar (*p* = 0.96; Figure 1D).

**Figure 1:**
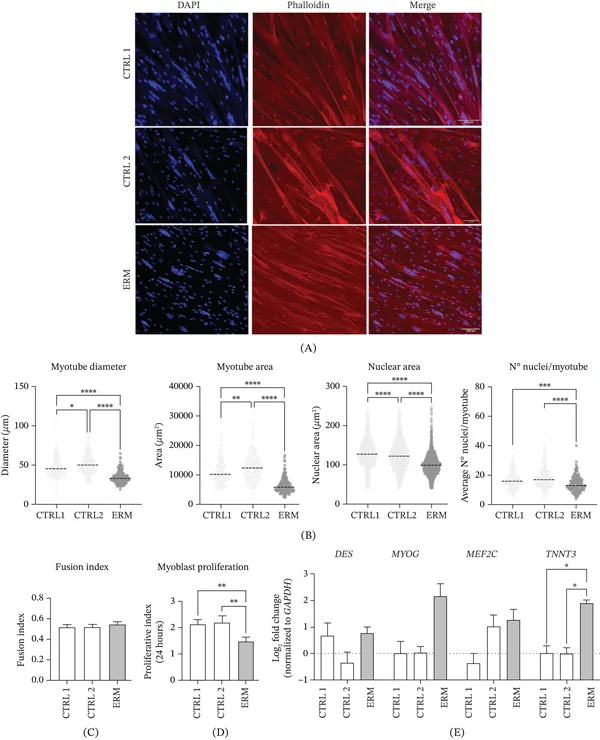
Morphological characterization of myotubes from immortalized cells derived from two healthy controls and the individual with exertional rhabdomyolysis carrying the heterozygous p.T4288_A4290dup RyR1 variant. (A) Immunofluorescence staining showing the nuclear (DAPI, blue) and actin (phalloidin, red) cytoskeletal organization of myotubes derived from healthy controls (CTRL1 and CTRL2) and the ERM individual. Images were acquired with a Nikon Ti2 widefield microscope equipped with a Nikon CFI Plan Apo Lambda 20x air objective (NA = 0.75). Multichannel fluorescent images were acquired with a Photometrics Prime 95B sCMOS camera, and image acquisition was controlled via Nikon NIS‐Elements software.). White bars, 100 *μ*m. (B) Average measurements of myotube transverse diameter (*μ*m), myotube area (*μ*m^2^), nuclear area (*μ*m^2^) and number of nuclei per myotube in CTRL (white symbols), and ERM (grey symbols) myotubes. Each symbol represents one myotube (for diameter, area, and nuclei/myotube) or one nucleus (nuclear area). The horizontal line indicates the median. Statistical analysis was performed using one way ANOVA followed by Tukey′s multiple comparison test.  ^∗^adjusted *p* < 0.05,  ^∗∗^adjusted *p* < 0.01,  ^∗∗∗^adjusted *p* < 0.001,  ^∗∗∗∗^adjusted *p* < 0.0001. (C) Quantification of fusion index in CTRL1 and CTRL2 (white bar) and ERM (grey bar) myotubes. The fusion index was calculated as the percentage of nuclei located within one myotube, relative to the total number of nuclei. Data are presented as mean ± SEM from 23 CTRL1 images, 20 CTRL2 images, and 34 ERM images. Statistical analysis was performed using one way ANOVA followed by Tukey′s multiple comparison test. (D) Proliferation index of immortalized myoblasts from CTRL1 and CTRL2 (white bars) and ERM (grey bar). The proliferation index was calculated as the ratio of the absorbance at 490 nm at 24 h to that at time 0 (ODT24/ODT0) as detailed in the Methods section. Each bar represents the mean of 7 independent measurements per cell line performed as technical duplicates. Data are presented as mean ± SEM. Statistical significance was determined using the Kruskal–Wallis test followed by an uncorrected Dunn′s multiple comparisons test for post hoc analysis;  ^∗∗^ *p* < 0.01. (E) Relative expression (log_2_ fold change) of the genes encoding the myotube maturation markers *DES*, *MYOG*, *MEF2C*, and *TNNT3* in CTRL1 and CTRL2 (white bar) and ERM (grey bar) myotubes. Each bar shows the mean of six independent qPCR experiments performed in technical duplicates. The mean values of CTRL1 and CTRL2 were set to 1 (dashed horizontal line). Gene expression was normalized to the housekeeping gene *GAPDH*. Data are presented as mean ± SEM. Statistical significance was determined using the Kruskal–Wallis test followed by an uncorrected Dunn′s multiple comparisons test for post hoc analysis; adjusted  ^∗∗^ *p* < 0.05.

These morphometrical differences between the ERM and the control cell lines may indicate a change in maturation brought about by altered calcium homeostasis linked to the presence of RyR1 variant; we therefore evaluated whether the cell lines show changes in the expression levels of myotube maturation markers. The expression level of the late maturation marker troponin T (*TNNT3*) was more than 3 times higher in the ERM myotubes compared with the two healthy control myotubes (the adjusted *p* value = 0.0105 for ERM versus CTRL1 and ERM versus CTRL2, respectively. Kruskal–Wallis test followed by Dunn′s multiple comparison test for post hoc analysis) (Figure 1E), whereas the expression levels of the early maturation markers desmin (*DES*) and myogenin (*MYOG*) and of the middle maturation marker Myocyte Enhancer Factor 2C (*MEF2C*) were not significantly different between the ERM and the two control cell lines (Figure 1E).

In conclusion the results obtained by analyzing the morphological characteristics of myotubes and the expression of differentiation markers, point to a potential impact of the RyR1 variant on the maturation properties of myotubes.

### 3.2. Enhanced Excitation Contraction Coupling in Myotubes From the ERM Individual

Calcium signaling plays an important role in myogenesis [31, 32] and *RYR1* mutations significantly affect the amount of calcium released via the RyR1 calcium channel. We therefore investigated whether the presence of the RyR1 variant c.T4288_A4290dup in ERM‐derived myotubes affects calcium homeostasis. Myotubes were loaded with the fluorescent calcium indicator Fura‐2 and stimulated with different concentrations of KCl to mimic depolarization, or 4‐chloro‐m‐chresol (4‐cmc) to pharmacologically activate the RyR1. Figures 2A,B show representative calcium transients induced by 60 mM KCl and 750 *μ*M 4‐cmc, respectively, in ERM (straight line) and CTRL1 myotubes (dot‐dashed line). The peak calcium released by both KCl and 4‐cmc was significantly higher in the ERM myotubes than control myotubes. The mean (±SEM) ΔF fluorescent ratio at 60 mM KCl was 0.60 ± 0.03*n* = 63 for ERM, 0.36 ± 0.03*n* = 27 for CTRL1 and 0.31 ± 0.03*n* = 18 for CTRL2 (*p* < 0.0001 one way ANOVA followed by uncorrected Fisher′s post hoc test) (Figure 2C); the mean (±SEM) ΔF fluorescent ratio at 750 *μ*M 4‐cmc was 0.66 ± 0.05*n* = 47 for ERM, 0.33 ± 0.06*n* = 19 for CTRL1 (*p* = 0.0012 one way ANOVA followed by uncorrected Fisher′s LSD post hoc test) and 0.47 ± 0.07*n* = 24 for CTRL2 (*p* = 0.048 one way ANOVA followed by uncorrected Fisher′s LSD post hoc test) (Figure 2D). The dose–response curves to 4‐cmc confirmed that the ERM myotubes show a shift to lower agonist concentrations. The EC_50_ for 4‐cmc was 144.7 ± 55, 207.9 ± 47.6, and 180.0 ± 76.6 for ERM, CTRL1, and CTRL2, respectively (Figure 2E,F). Although the EC_50_ to KCl was similar in myotubes from ERM and CTRL2 (17.6 ± 2.1 and 13.3 ± 2.2, respectively) but higher in myotubes from CTRL1 (27.6 ± 2.6). ERM‐derived myotubes also showed a small increase in the resting [Ca^2+^]_I_ (mean ± SEM) 0.84 ± 0.01 (*n* = 1164) in ERM, 0.63 ± 0.01 (*n* = 244) in CTRL1 and 0.65 ± 0.01 CTRL2 myotubes (one way ANOVA followed by uncorrected Fisher′s LSD post hoc test *p* < 0.0001) (Figure 3G) whereas the total amount of releasable calcium in the intracellular stores was similar in ERM and control myotubes (Figure 2H).

**Figure 2:**
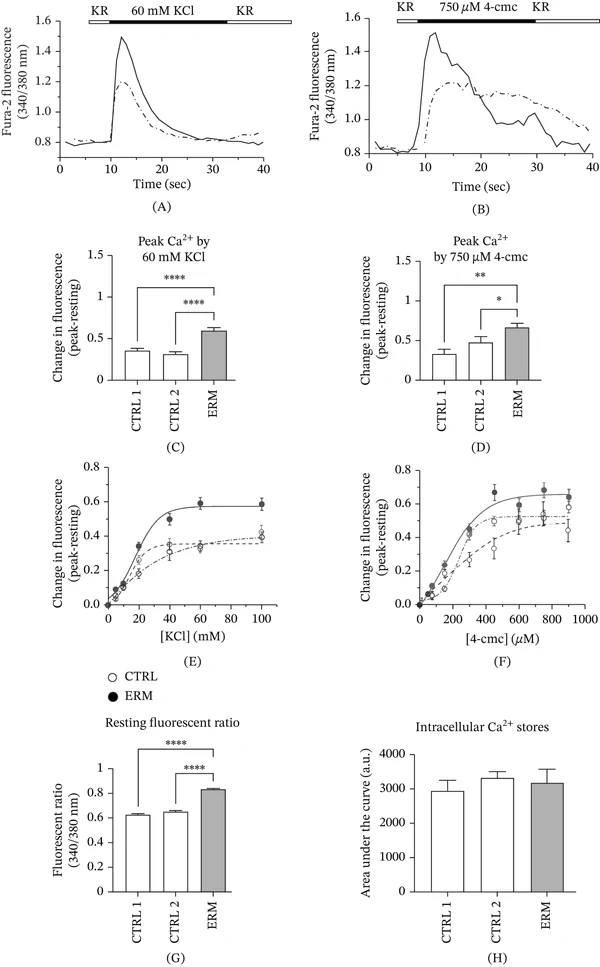
Calcium homeostasis in control and ERM myotubes. (A,B) Representative traces showing time‐dependent changes in fura‐2 fluorescence (340/380 nm) after the application of (A) 60 mM KCl and (B) 750 *μ*M 4‐chloro‐m‐cresol (4‐cmc). The dashed lines indicate CTRL1 myotubes and solid black lines indicate ERM myotubes (KR, Krebs–Ringer solution). (C,D) ΔF fura‐2 fluorescence (peak fluorescence–resting fluorescence) induced by (C) 60 mM KCl and (D) 750 *μ*M 4‐cmc in CTRL1 and CTRL2 (white bars) and ERM (grey bars) myotubes. Data are presented as mean ± SEM; for (C) 60 mM KCl *n* = 27 for CTRL1, *n* = 18 for CTRL2 and *n* = 63 for ERM myotubes and for (D) 750 *μ*M 4‐cmc *n* = 19 for CTRL1, *n* = 24 for CTRL2 and *n* = 47 for ERM myotubes. (E, F) Dose–response curves of myotubes to (E) KCl and (F) 4‐cmc. Dashed dot lines (‐.‐.‐) represent CTRL1 myotubes, dashed lines (‐‐‐‐) represent CTRL2 myotubes and solid lines (—) represent ERM myotubes. Each symbol represents the mean ΔF (± SEM) of 15–60 myotubes. Dose–response curves were generated using the Origin software. (G) Mean (±SEM) resting [Ca^2+^]_i_ expressed as fura‐2 fluorescence ratio (340/380 nm). The white bars represent CTRL1 (*n* = 244 myotubes) and CTRL2 (*n* = 165 myotubes), and the grey bar represents ERM (*n* = 1164 myotubes). (H) Quantification of intracellular Ca^2+^ stores, determined by calculating the area under the curve after application of 500 nM thapsigargin and 1 *μ*M ionomycin. The white bars represent (mean ± SEM) CTRL1 (*n* = 13 myotubes) and CTRL2 (*n* = 14 myotubes), and the grey bar represents ERM (*n* = 15 myotubes). Statistical comparison was performed using the one‐way ANOVA followed by the uncorrected Fisher′s LSD for post hoc analysis.  ^∗^ *p* < 0.05,  ^∗∗^ *p* < 0.01, and  ^∗∗∗∗^ *p* < 0.0001.

**Figure 3:**
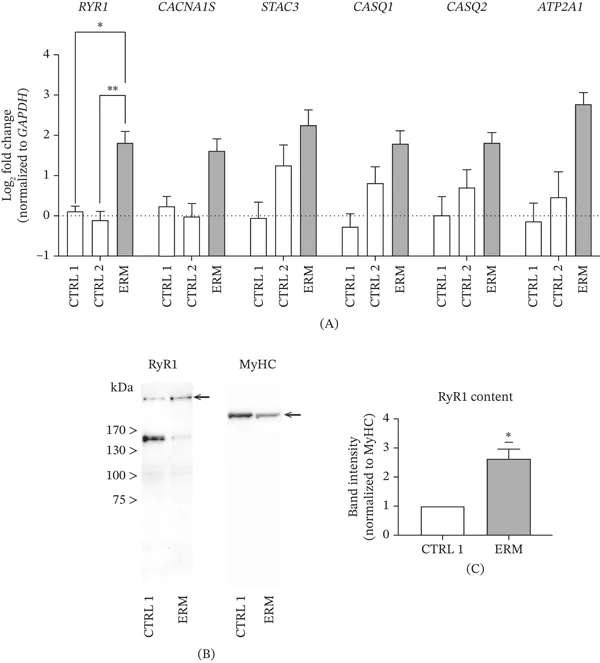
The expression of the transcripts encoding *RYR1* and RyR1 protein content are increased in ERM myotubes compared to control myotubes. (A) Relative expression levels of transcripts encoding ECC proteins (*RYR1*, *CACNA1S*, *STAC3*, *CASQ1*, *CASQ2*, and *ATP2A1*) were assessed in CTRL1 and CTRL2 myotubes (white bars) and ERM myotubes (grey bars) by qPCR. Gene expression was normalized to the housekeeping gene *GAPDH*. Each bar represents the mean of at least six independent qPCR experiments performed in technical duplicates. The mean values of CTRL1 and CTRL2 were set to 1 (dashed horizontal line). Data are presented as mean ± SEM. Statistical analysis was performed using the Kruskal–Wallis test followed by the Dunn′s multiple comparisons test for post hoc analysis. Corrected *p* values are shown, where  ^∗^ *p* < 0.05,  ^∗∗^ *p* < 0.01. (B) Representative western blot showing RyR1 protein content in total homogenates (30 *μ*g protein/lane) prepared from CTRL1 (left lanes) and ERM (right lanes) myotubes. The arrow indicates the immunopositive band corresponding to the RyR1 protomer. After detection of RyR1, the blot was stripped and reprobed with an antibody recognizing all myosin heavy chain isoforms (MyHC, arrow) used as a loading control (6% SDS‐PAG). (C) Bar graph shows RyR1 band intensity normalized to myosin heavy chain, with CTRL1 set to 1. White and grey bars represent CTRL1 and ERM myotubes, respectively. Data are presented as mean ± SD*n* = 3 blots. Statistical significance was determined using an unpaired *t*‐test  ^∗^ *p* < 0.05.

Because of the increased levels in transcription factors related to maturation and the increased levels of calcium release following agonist stimulation, we also investigated whether ERM myotubes show changes in the expression levels of transcripts encoding ECC proteins. Figure 3A shows that the relative expression level of *RYR1* normalized to the housekeeping gene *GAPDH* was significantly higher in ERM myotubes compared to controls (adjusted *p* value = 0.028 for ERM vs. CTRL1 and *p* = 0.0035 for ERM vs. CTRL2 Kruskal–Wallis test followed by Dunn′s multiple comparison test for post hoc analysis) (Figure 3A), whereas the expression levels of all other transcripts encoding ECC genes were not significantly different between the ERM myotubes and myotubes from the two control cell lines. The increased content of RyR1 was also confirmed by western blotting on total homogenates prepared from ERM and CTRL1 myotubes. As shown in Figure 3B the high molecular weight immunopositive band corresponding to the RyR1 protomer (left panel) is more pronounced in ERM than CTRL1 homogenates. Band intensities were normalized for myosin heavy chain content (right panel, loading control). Figure 3C shows that the mean (±SEM, *n* = 3) band intensity of the RyR1 was significantly higher in ERM than CTRL1 myotubes (2.64 ± 0.32 vs. 1.0, *p* = 0.015 unpaired *t* test) confirming that the level of both the *RYR1* transcript and RyR1 protein are increased in ERM myotubes.

In conclusion the characterization of ECC in myotubes provides evidence of enhanced calcium release and increased expression of the transcript encoding *RYR1* in myotubes from the ERM individual substantiating the finding that they exhibit enhanced maturation characteristics,

### 3.3. Calpain Activity Is Increased in ERM‐Myotubes Following Membrane Depolarization

The results obtained so far indicate a potential change in myotube maturation/development in the cell line derived from the ERM individual, together with alterations of calcium homeostasis. However, they do not shed any light into the mechanism whereby the RyR1 variant leads to rhabdomyolysis and muscle breakdown in Pt ERM_01. In the next series of experiments, we examined the expression of calpain, a calcium dependent protease that could be implicated in muscle breakdown. Since the two control cell lines were very similar in their maturation properties and calcium homeostasis, all subsequent experiments were conducted only on CTRL1. Fifteen genes encoding calpain isoforms have been identified in the human genome, including the ubiquitously expressed calpain‐1 which is activated by *μ*M calcium and plays a role in myofibrillar protein and junctophilin degradation, and myotube atrophy and calpain‐2 which is activated by mM calcium and plays a role in the calcium‐dependent repair of damaged myotube membranes [33–37]. Calpain‐3 is only expressed in skeletal muscle where it plays a role in sarcomere assembly, triad maintenance, and renewal of the satellite cell compartment [36]. qPCR experiments revealed that *CAPN2* is expressed at very low levels in the myotubes obtained from the immortalized human cell lines, so we did not investigate it further. We assessed whether there are differences in *CAPN1* and *CAPN3* transcript levels between CTRL1 and ERM myotubes under physiological conditions (37°C) or excessive heat (42°C) and at rest or after membrane depolarization. The levels of *CAPN1* transcripts were similar in both cell lines whether the cells were incubated at 37°C or 42°C (Figure 4A). On the other hand, the transcript levels of *CAPN3* were threefold higher in myotubes from the ERM individual compared with CTRL1 when the cells were incubated at 37°C (the expression level of *CAPN3* was 1.0 ± 0.08 and 3.1 ± 1.2 in CTRL1 and ERM, respectively, *n* = 6*p* = 0.0022, Student′s *t* test) (Figure 4B) and its expression level increased further when ERM cells were incubated at 42°C for 4 h (the expression level of *CAPN3* in CTRL1 and ERM myotubes incubated at 42°C was 1.2 ± 0.4 and 8.0 ± 3.1, respectively, *n* = 6*p* = 0.0022, Student′s t test) (Figure 4B). We also checked whether membrane depolarization affects *CAPN3* transcript levels, but no significant differences were found in its expression levels at any time points neither in control, nor ERM myotubes (Figure 4C).

**Figure 4:**
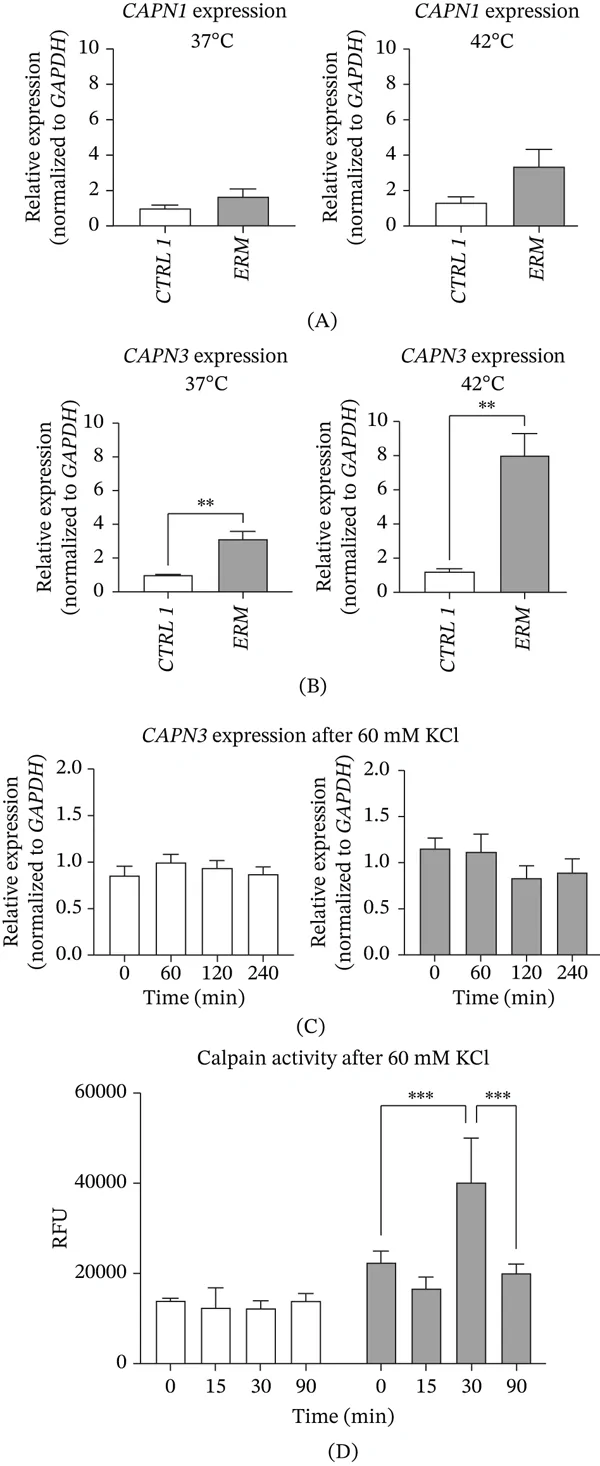
*CAPN3* expression and depolarization‐induced calpain activity, are increased in ERM myotubes compared with CTRL1. (A) Relative expression of *CAPN1* transcript in control (white bars) and ERM myotubes (grey bars) incubated at 37°C (left panel) and at 42°C (right panel) for 240 min. Data are presented as mean (±SEM) from six independent experiments. (B) Relative expression of *CAPN3* transcript in CTRL1 (white bars) and ERM myotubes (grey bars) incubated at 37°C (left panel) and at 42°C (right panel) for 240 min. Results are plotted as relative expression normalized to the expression of the housekeeping gene *GAPDH* (mean fold change ±SEM, from six independent experiments). (C) Time course of *CAPN3* expression following membrane depolarization with 60 mM KCl. Relative transcript levels are expressed as fold change relative to levels observed in cells incubated with Krebs–Ringer alone for 5 min at 37°C. Left panel (white bars) CTRL1 myotubes, right panel (grey bars) ERM myotubes. Data are presented as mean ± SEM from six independent experiments. (D) Quantification of total calpain activity of myotubes following membrane depolarization induced by 60 mM KCl in control (CTRL1; white bars) and ERM (grey bars) myotubes. Data are presented as relative fluorescence units (RFU). Each bar indicates mean ± SD from three independent experiments performed as duplicates. For panels A and B, statistical comparisons were performed using the Student′s *t* test  ^∗∗^ *p* < 0.01. Panel D statistical comparisons was performed using the ANOVA followed by Tukey′s post hoc test.  ^∗∗∗^ *p* < 0.001.

Since in ERM myotubes the depolarization‐induced Ca^2+^ transient is significantly larger than that in CTRL1 myotubes and calpain activity is calcium dependent, we next investigated if membrane depolarization is accompanied by the activation of calpain′s enzymatic activity. Figure 4D shows that in ERM‐derived myotubes (grey bars) but not in CTRL1 myotubes (white bars), 30 min after membrane depolarization there is a transient increase in total calpain activity, which returns to resting levels by 90 min. The mean calculated calpain activity at *t* = 0 was 22496.5 ± 2485.2 RFU (*n* = 3) and peaked to 40155.3 ± 9882.9 RFU (*n* = 3) at *t* = 30 in ERM myotubes (Tukey′s multiple comparison test, adjusted *p* value = 0.001), whereas the activity in CTRL1 myotubes was 14008.3 ± 524.9 at *t* = 0 (*n* = 3) and 12280.3 ± 1709.2 at *t* = 30 (*n* = 3) in control myotubes.

In conclusion, the results obtained in this section indicate that, in vitro, myotubes derived from the ERM individual carrying the RyR1 variant c.T4288_A4290dup not only exhibit significantly higher levels of *CAPN3* transcripts than control myotubes (and that these levels are further increased when myotubes are incubated at 42°C), but also that ERM myotubes have a higher basal level of calpain activity, which increases significantly by prolonged membrane depolarization—a finding in line with the observed increased size of the calcium transient in myotubes harboring the RyR1 variant.

### 3.4. Proteomic Analysis of Muscle Biopsies

The results obtained so far show that there are substantial differences between the cell line immortalized from the ERM individual carrying the heterozygous RyR1 p.T4288_A4290dup and healthy controls; however, they have the limitation that the observations are limited to one biological sample and to myotubes. The latter may not faithfully reflect the status of mature muscle tissue. To this end, we obtained muscle biopsies from three males carrying the same heterozygous RyR1 p.T4288_A4290dup variant, including the same index case Pt ERM_01, as well as Pt ERM_02 and Pt ERM_03 (see section on Muscle Biopsies in the Materials and Methods for additional information) and age‐matched control males and subjected the muscle samples to mass spectrometry. PCA analysis of the proteomic data shows that the two cohorts can be separated and that the biopsies carrying the RyR1 variants (light blue dots/triangle) group together and are distinct from the control group biopsies (red dots/triangle) (Figure 5A) providing evidence that there are intrinsic differences in the overall protein composition between the two cohorts.

**Figure 5:**
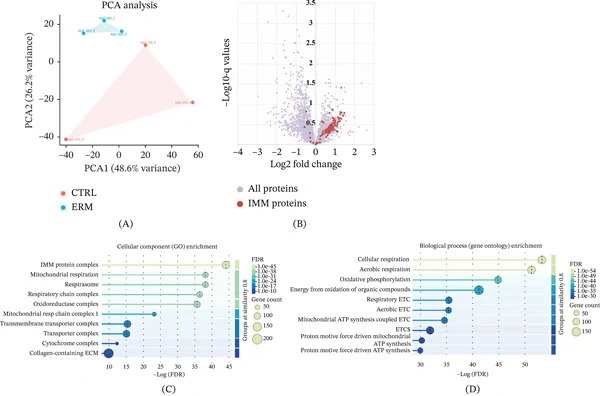
Proteomic analysis of muscle biopsies from individuals carrying the heterozygous RyR1 p.T4288_A4290dup variant versus biopsies from age and sex‐matched controls. (A) Principal component analysis (PCA) showing separation of the overall protein content between muscle biopsies from individuals carrying the heterozygous RyR1 p.T4288_A4290dup variant (blue symbols and triangle) and age and sex‐matched control biopsies (red symbols and triangle). (B) Volcano plot analysis comparing the relative protein expression between the two cohorts. A total of 2949 proteins were included in the analysis and threshold values of log2FC > 1 or log2FC ≤ 1 were taken into consideration. Red dots represent proteins that are constituents of the inner mitochondrial membrane (IMM). (C,D) Lollipop plots showing enriched Gene Ontology (GO) terms identified by STRING rank‐based enrichment analysis. The top TEN hits with lowest False Discovery Rate (FDR) were selected. (C) Cellular component (GO) enrichment. (D) Biological process (GO) enrichment. Clusters were ranked according to (‐axis), and enriched terms are displayed on the ‐axis. Circle size indicates the number of proteins associated with each term, while color represents the FDR of the enrichment. Figures were directly exported from STRING.

No individual protein or components of the inner mitochondrial membrane (IMM) passed the significance threshold on their own, however, as a cluster they exhibit a coordinated positive shift in fold change (Figure 5B), suggesting small but consistent expression changes. To explore whether differentially expressed proteins consist of functionally related groups of proteins we performed a log_2_FC rank‐based enrichment analysis using STRING [38]. The most significantly enriched clusters of GO terms (among cellular components Figure 5C and biological processes Figure 5D) clearly point towards changes in aerobic respiration with remodeling of the IMM. In addition, 33 proteins showed a significant decrease in content in the muscle biopsies from individuals carrying the RyR1 p.T4288_A4290dup variant compared with controls (Table 1).

We also investigated whether the relative content of proteins involved in ECC and calcium homeostasis was different in control versus ERM biopsies. To this end, we calculated the mean relative content of the selected protein in the biopsies from control individuals (values in brackets, Table 2) and set this value to 100%. Subsequently, we calculated the relative % content of each protein in the biopsy from each ERM individual, as well as the mean relative % content in the ERM group as a whole. As shown in Table 2, the biopsies from the control group were homogeneous, showing ≈ 10% variability in the content of ECC proteins. On the other hand, the biopsies from the ERM individuals showed a large variability (> 50%) in the content of many proteins involved in ECC. Since the content in ECC proteins varies between fast twitch and slow twitch muscles, we also investigated the relative content of myosin heavy chain isoforms to determine whether the observed variability could be due to the higher composition of fast twitch fibers (which express higher levels of RyR1, Cav1.1, Casq1, and SERCA1) or slow twitch fibers (which express higher levels of Casq2 and SERCA2). Interestingly the muscle biopsy from individual Pt ERM_01 contained a higher proportion of fast than slow twitch fibers, the muscle biopsy from Pt ERM_02 was mostly composed of slow twitch fibers whereas the muscle biopsy from Pt ERM_03 was mostly composed of fast twitch fibers.

**Table 2:** Relative content of proteins involved in ECC and calcium homeostasis in muscle biopsies from healthy controls and ERM individuals.

Protein	Ctrl 1/^a^CTRL mean	Ctrl 2/^a^CTRL mean	Ctrl 3/^a^CTRL mean	Ctrl mean	Pt ERM_01/^a^CTRL mean	Pt ERM_0 2/^a^CTRL mean	Pt ERM_03/^a^CTRL mean	MEAN ERM^/a^CTRL mean
RyR1	108.9%	95.9%	95.3%	100%	102.5%	**83.9%**	**150%**	**112.1%**
	(35936)	(31657)	(31461)	(33018)	(33842)	(27706)	(49524)	
Cacna1s	1.13%	88.7%	0.98%	100%	**75.1%**	**77.6%**	**155.5%**	**102.7%**
	(3417)	(2687)	(2987)	(3030)	(2275)	(2353)	(4713)	
Stac3	103.4%	92.5%	104.1%	100%	90.5%	**84.2%**	**138.4%**	**104.4%**
	(1223)	(1093)	(1231)	(1182)	(1070)	(995)	(1638)	
Casq1	89.9%	97.1%	113.1%	100%	**84.5%**	**66.2%**	**192.1%**	**114.3%**
	(84148)	(90894)	(105875)	(93639)	(79129)	(62037)	(179859)	
Casq2	105.8%	98.7%	95.5%	100%	**69.2%**	**313.3%**	**58.1%**	**146.9%**
	(5896)	(5503)	(5323)	(5574)	(3858)	(17462)	(3239)	
Serca1	94.3%	90.6%	115.1%	100%	97.8%	**58.7%**	**194.7%**	**117.1%**
	(400157)	(384485)	(488369)	(424337)	(414863)	(248924)	(826167)	
Serca2	111.8%	96.1%	92.2%	100%	**44.7%**	**172.8%**	**3.6%**	**73.7%**
	(99303)	(85290)	(81929)	(88841)	(39730)	(153560)	(3237)	
JP‐45	107.4%	101.2%	91.3%	100%	108.4%	90.5%	**191.1%**	**130.0%**
	(3132)	(2950)	(2662)	(2915)	(3160)	(2638)	(5570)	
Junctophilin 1	106.2%	97.8%	96.0%	100%	117.3%	**69.1%**	**157.1%**	**114.5%**
	(2802)	(2579)	(2533)	(2638)	(3094)	(1823)	(4154)	
Stim 1	97.2%	110.5%	92.3%	100%	107.9%	108.6%	106.1%	**107.5%**
	(777)	(883)	(738)	(799)	(862)	(868)	(848)	
MyHC 2A	88.1%	110.0%	101.9%	100%	117.6%	89.5%	**28.1%**	**78.4%**
	(755193)	(942720)	(874044)	(857319)	(1007940)	(767434)	(240819)	
MyHC‐slow	107.5%	106.4%	86.1%	100%	**63.9%**	**144.6%**	**3.8%**	**70.8%**
	(3001964)	(2970242)	2405640	(2792615)	(1785653)	(4039988)	(107427)	
MyHC‐emb	105.3%	87.1%	107.6%	100%	88.1%	**64.6%**	**137.4**%	**96.7%**
	(30644)	(25345)	(31296)	(29095)	(25635)	(18813)	(39984)	
MyHC‐2X	103.9%	92.2%	103.9%	100%	96.6%	**68.1%**	**123.1%**	**95.9%**
	(4016122)	(3563444)	(4017664)	(3865743)	(3736429)	(2633623)	(4757806)	
MyHC‐2B	95.8%	100.2%	104.0%	100%	119.8%	**75.8%**	140.4%	**112.0%**
	(56637)	(59231)	(61497)	(59122)	(70804)	(44801)	(83011)	

*Note:* Values in bold show a change in relative protein content > 15%.

^a^The mean (relative protein content) of each indicated protein in muscle biopsies from Control 1, Control 2 and Control 3 was averaged and set as 100%.

In conclusion mass spectrometry analysis of muscle biopsies from ERM and controls revealed that the two cohorts could be clearly separated from each other and that ERM biopsies were enriched in proteins involved in aerobic respiration and mitochondria electron transport chain proton transport. Intriguingly the fiber type composition of the muscles of the ERM group was more heterogeneous than that of the control group.

## 4. Discussion

In the present work we provide evidence that myotubes obtained from an immortalized muscle cell line established from an individual with ERM and carrying the heterozygous p.T4288_A4290dup RyR1 variant are different from myotubes obtained from immortalized muscle cell lines established from two healthy controls. In particular, the morphometric characteristics of the cells showed significant size differences, as did the expression of the late maturation marker *TNNT3* as well as of the *RYR1* gene. In addition, the presence of the RyR1 variant was accompanied by larger depolarization‐induced calcium transients. In vitro, prolonged membrane depolarization was accompanied by the activation of calcium‐dependent calpain activity which was substantially higher in the cells carrying the RyR1 variant compared to controls.

There are several limitations to this study, which hamper us from making generalized conclusions regarding the RyR1 p.T4288_A4290dup variant: firstly, we characterized one cell line carrying the heterozygous RyR1 duplication and secondly, whole exome analysis on the index case was not performed so he may potentially carry other genetic variants that affect Ca^2+^ homeostasis. However, this same RyR1 p.T4288_A4290dup was already reported by Levano et al. [20] in a subject with the MHS phenotype, by Dlamini et al. [7] in a subject with ERM and by Sambuughin et al. [21], in a subject with ERM and malignant hyperthermia. Genetic investigation on the individual analysed by Sambuughin et al. [21] revealed that he also carried the p.S1342G plus the p.A1352G RyR1 variants. Although p.S1342G was reported as being a polymorphism detected in unaffected control samples ([20, 39]. Allelic frequency 0.008672 https://gnomad.broadinstitute.org/), the p.A1352G RyR1 variant has a reported frequency of 2.7% in the African American population (allelic frequency in African/African American ancestry 0.02221; https://gnomad.broadinstitute.org/) and is also present in MHN individuals [21]. Thus, it is likely that the RyR1 p.T4288_A4290dup is responsible for the ERM phenotype and the changes in the functional properties of the RyR1 calcium channel characterized in the present report are due to the presence of the small duplication in exon 91.

### 4.1. Calcium Homeostasis in Myotubes Carrying the RyR1 P.T4288_A4290dup Variant

In the context of calcium homeostasis, myotubes carrying the RyR1 p.T4288_A4290dup were physiologically different from other myotubes carrying gain of function RyR1 variants associated with the MHS phenotype that have previously been characterized such as the p.R163C and the p.V2168M variants [26, 40]. In fact, the amount of calcium released after membrane depolarization and/or pharmacological activation of RyR1 was significantly higher in ERM myotubes than in myotubes from the two control cell lines, even though their intracellular calcium stores were similar. Though we cannot completely exclude that the higher Ca^2+^ peak observed after KCl and 4‐cmc stimulation is not due at least in part, to the higher content of RyR1 in ERM myotubes, the 3 amino acid duplication is located between RyR1 residues T4288 and A4290, a region that has been reported to act as a negative regulator of RyR1 calcium channel opening. In fact, Du et al. [41] showed that expression of a RyR1 construct carrying a deletion of residues Δ4274–4535 in dyspedic myotubes causes an increase in the maximal amplitude of electrically evoked Ca^2+^ transients and a shift in voltage gated Ca^2+^ release, without affecting orthograde or retrograde RyR1 signals of ECC. Thus, removing the region contained between residues 4274–4535 or changing its structure by inserting a small duplication, most likely interferes with a domain involved in the negative regulation of Ca^2+^ release. An interesting observation from the current investigation relates to the small but significant increase in the resting [Ca^2+^] observed in ERM versus control myotubes. This may be due to several factors, including an increased calcium influx linked to the presence of the RyR1 variant [42]. Indeed, in the case of MHS‐causing mutations studies have shown that they are accompanied by an increase the resting [Ca^2+^], but this does not result in smaller calcium stores since myotubes can activate compensatory mechanisms to maintain the total calcium load of the sarcoplasmic reticulum [42, 43]. Another explanation may relate to the fact that ERM myotubes are more differentiated than control myotubes. Recently Benarroch et al. [44] showed that MyoD transduced fibroblasts are similar to primary myotubes but never achieve the same degree of differentiation and in a previous study on primary myotubes and myotubes obtained by MyoD transduction of fibroblasts, we showed that resting [Ca^2+^] is significantly higher in primary myotubes compared with MyoD‐myotubes [45]. Thus, a higher degree of differentiation could result in changes of the resting [Ca^2+^] because of differentially expressed proteins involved in calcium regulation.

As far as the characterization of only one immortalized cell line from the ERM individual, we would like to point out that we performed morphometric, calcium homeostasis, and transcriptional analysis on cell lines from two control individuals and these were very similar to each other and significantly different from the cell line derived from the ERM individual. These results raise the possibility that the differences in size, nuclei, and differentiation characteristics observed in the myotubes derived from the ERM individual reflect an intrinsic property of the myotubes carrying the RyR1 duplication. As to the mechanism linking calcium homeostasis and muscle cell differentiation, myoblast fusion is calcium‐dependent and differentiation is inhibited by calcium depletion. [46]. Furthermore, incubation of C2C12 myoblasts with nifedipine (a blocker of the DHPR L‐type calcium channel) and dantrolene (an inhibitor of the RyR1) inhibits differentiation into myotubes and inhibits the expression of differentiation markers [32]. These results support a role of RyR1 and ECC in myotube differentiation. As to the effect of the *RYR1* variant on myoblast proliferation, our experiments do not provide direct evidence that the altered RyR1‐mediated Ca^2+^ signaling leads to a reduced proliferation rate. Future experiments aimed at determining whether there is a direct correlation between altered calcium homeostasis, myoblast proliferation, myotube size and degree of differentiation will clarify this matter.

### 4.2. Calpains in ERM Myotubes

A novel finding of this report deals with the higher transcript levels of *CAPN3* in mutant myotubes and, more importantly, the increased enzymatic activity of calpain observed in mutant myotubes after membrane depolarization. Though the exact role of calpains in skeletal muscle physiology is far from understood, these calcium‐dependent proteases play an important role in myotube fusion and differentiation by promoting the degradation of cytoskeletal proteins and filamin [47, 48]. Such a finding is compatible with our observations that ERM myotubes show a higher degree of differentiation and higher levels of *CAPN3* transcript expression. As to its enzymatic activity, an increased myoplasmic calcium concentration in muscle fibers and cultured myotubes (from 200 nM to 1‐2 *μ*M) can activate the autocatalytic activity of calpain‐3 leading to its translocation from the M‐band (where it is in the inactive form) to the cytosol, where it is enzymatically active [49–52]. In the M‐band calpain is linked to titin and its detachment may lead to M‐band destabilization, whereas the translocation of the catalytic portion to the cytosol leads to digestion of sarcolemmal proteins including spectrin and talin as well as junctophilin, the latter protein providing an important role in the maintenance of the structural integrity of triads [37]. These events have been linked to sarcolemmal remodeling; however if they occur repeatedly as for example following repeated bouts of eccentric exercises, they may cause loss of sarcolemmal integrity [50], providing a potential link to rhabdomyolysis.

### 4.3. Proteomic Analysis of Muscle Biopsies From ERM Versus Control Individuals

To our knowledge this is the first study on the proteomic characterization of human muscle biopsies from patients with RyR1 variants, though the small number of samples precludes us from drawing definitive conclusions. Compared with the large changes in protein content revealed by mass spectrometry analysis of muscles from mice carrying recessive *Ryr1* variants [29], the human muscle biopsies from rhabdomyolysis individuals were more similar to control muscle biopsies. However, the muscle biopsies from the control individuals were more similar to each other, containing both fast and slow twitch muscle fibers, whereas the muscle biopsies from the ERM individuals were skewed and contained either a greater proportion of fast twitch (Pt ERM_03) or slow twitch muscle fibers (Pt ERM_02) as shown by the content of myosin heavy chain isoforms, SERCA and calsequestrin isoforms. As to specific changes in protein content, there were 33 proteins that were depleted in the muscles of ERM patients, but no changes in the content of proteins involved in skeletal muscle ECC were found. Thus, the observed discrepancy between the increased content of RyR1 in myotubes but not in mature fully differentiated muscles is probably related to the fact that mature muscle fibers contain much higher levels of proteins involved in ECC compared with myotubes. Upon differentiation and fusion of myotubes into mature muscle tissue, the amount of RyR1 protein increases dramatically and most likely reaches a saturating level whereby no additional calcium channels are needed for muscle function and can be inserted into the sarcoplasmic reticulum membrane. The proteomic analysis and biochemical/physiological data presented in this study suggest that under normal resting conditions, fully differentiated muscles can cope with the physiological changes brought about as a consequence of the RyR1 duplication, but under stressful conditions, such as intense exercise and hot conditions the fine balance is lost, initiating a cascade of events which ultimately result in myofiber death and rhabdomyolysis.

In conclusion, this is the first study characterizing the functional effect of a RyR1 variant linked to the ERM phenotype. Overall, these results support the role of the RyR1 p.T4288_A4290dup variant as a cause of ERM and these findings are also relevant for diagnostic and prognostic reasons, as currently this variant is not reported by diagnostic laboratories. Additional studies, particularly the creation of a mouse model knocked in for the RyR1 p.T4288_A4290dup characterized in the present report, would provide further evidence to firmly confirm our observation.

The results of the present study also provide potential mechanistic insight into the link between the RyR1 p.T4288_A4290dup and enhanced athletic abilities of the variant carriers as well as the predisposition to exercise‐induced rhabdomyolysis. We hypothesize that the gain of function variants leads to enhanced ECC and larger amounts of calcium released from the sarcoplasmic reticulum, facilitating muscle contraction, promoting differentiation, and potentially leading to muscle hypertrophy This increased calcium release however, will (i) activate the SERCA pumps to a greater extent during exercise and (ii) will activate calcium‐dependent muscle enzymes such as calpains. The combination of these events could lead to muscle breakdown and rhabdomyolysis if high intensity exercise and sport training are performed at high levels and especially during hot ambient temperatures.

## 5. Conclusions

This is the first report in which the functional properties of a RyR1 variant (the RyR1 p.T4288_A4290dup) associated with human rhabdomyolysis are characterized. By characterizing an immortalized muscle cell line carrying the variant, our results show that it causes changes in the morphological characteristics of myotubes, leading to more mature cells and increased expression of the transcript encoding *RYR1* and *CAPN3*. The RyR1 duplication is also accompanied by changes in the proteomic composition of mature muscles. We hypothesize that the gain‐of‐function RyR1 p.T4288_A4290dup variant leads to enhanced ECC and larger amounts of calcium released from the sarcoplasmic reticulum, facilitating muscle contraction, promoting differentiation, and potentially leading to muscle hypertrophy.

## Funding

This project was supported by the Swiss National Science Foundation (SNSF) (Grant No. 310030_231328); a grant from the ZNM, the SwissLife Foundation (Grant No. 1494), the Chinese Scholarship Council (Grant No. 202308410171), and the Japan Society for the Promotion of Science, Japan Society for the Promotion of Science and the FAR (Fondo di Ateneo per la Ricerca) University of Ferrara.

## Ethics Statement

This study was conducted in accordance with the Declaration of Helsinki (1964).

## Conflicts of Interest

The authors declare no conflicts of interest.

## Supporting information


**Supporting Information** Additional supporting information can be found online in the Supporting Information section. File S1: The Supporting Information section contains a table showing the sequence of the primers used for qPCR.

## Data Availability

The data that support the findings of this study are openly available in ProteomeXchange (PXD080541) at http://MSV000102354@massive‐ftp.ucsd.edu, Reference Number MSV000102354.
