# Supplementary material for: Functional Characterization of an Immortalized Human Muscle Cell Line Carrying the Heterozygous RyR1 p.T4288_A4290dup Associated With Rhabdomyolysis
Source: Hum Mutat. 2026 Oct 27;2026:8852225. doi: 10.1155/humu/8852225 (PMC13711367; doi:10.1155/humu/8852225)
Supplement: Supplementary file 1 [file HUMU-2026-8852225-s001.docx]

**Supplementary Table 1**: Sequence of primers used for qPCR

| Gene | Forward Primer | Reverse Primer |
| --- | --- | --- |
| *CACNA1S* | 5’-TGTCTTCCTGGGGGTCTTCA-3’ | 5’-GCTCCTTTGCTGCTCATTGG-3’ |
| *CAPN1* | 5’-CGCAGCCCGAGGATGTC-3’ | 5’-GGTCCTTGTAACCCAGGCTC-3’ |
| *CAPN3* | 5`-GCCCATCATCTTCGTTCGG-3` | 5`-CAGGACTCCAGTGTGAACCCC-3` |
| *CASQ1* | 5’-TGTTACTGATGCGGATAGCGT-3’ | 5’-CAGTGGGGCTGAAAGATGGT-3’ |
| *CASQ2* | 5’-CTTTCCTCTGCTCGTTGCCT-3’ | 5’-AGCACATCCTCAATCCAGTCC-3’ |
| *DES* | 5’-GGATGGGGAGGTCGTCAGT-3’ | 5’-CTGGGATGGAAGAAGGCTGG-3’ |
| *GAPDH* | 5’-GAGAAGGCTGGGGCTCATTT-3’ | 5’-ATGACGAACATGGGGGCATC-3’ |
| *MEF2C* | 5’-ACCA GAACCCCGCTTCTA-3’ | 5’-TACACACCTTACACGCCCAC-3’ |
| *MYOG* | 5’-GAACAAGCCTTTTCCGACCC-3’ | 5’-TAGAAGCGGGGTTCCTGGTA-3’ |
| *RYR1* | 5’-GATGCAAACATGCAGCGTCC-3’ | 5’-CTCTCCGTTCTGGCGATTGA-3’ |
| *ATP2A1* | 5’-CCGGACCAAGTTAAGCGGAA-3’ | 5’-ACAGGGTCTTCCCTTCCTCA-3’ |
| *STAC3* | 5’-AATGGTGGCGGGGGAAAATC-3’ | 5’-CCCGCTTCGTCTCCTTTCTG-3’ |
| *TNNT3* | 5’-ATTGACCAGGCCCAGAAGC-3’ | 5’-GGCCTTTCTGGCCTCTCTAC-3’ |
